# Supplementary material for: Duodenal Dual-Wavelength Photobiomodulation Improves Hyperglycemia and Hepatic Parameters with Alteration of Gut Microbiome in Type 2 Diabetes Animal Model
Source: Cells. 2022 Nov 3;11(21):3490. doi: 10.3390/cells11213490 (PMC9654760; doi:10.3390/cells11213490)
Supplement: Supplementary file 1 [file cells-11-03490-s001.zip › cells-1955216-supplementary.pdf]

Supplementary Method

Experiment:

Triangular 5-cm effective 10 mini-LED chips were attached in each two dimensions in 2.5 mm diameter and 6 cm in length of polyurethane catheter. Ten LED chips were placed with 5 mm apart in 5 cm in lengh of a light diffuser. The light power was measured with a calibrated detector (Newport 918D-ST-SL wand detector from 400 to 1100 nm; Newport Corp., CA, United States) and power meter (Newport 1936-R; Newport Corp., CA, United States). Catheter based radiofrequency ablation (RFA, 2.2cm length, 7W, 5 seconds Taewoong Medical) was applied for simulated duodenal mucosal resurfacing because hydrothermal RFA probe was not commercially available in Korea. Due to the severe burning effect of RFA for duodenum and early death in prior energy settings in SD rats, duration of RFA was limited to 5 seconds to reach target temperature 75-80 °C in catheter based RFA due to safety issue resulting in suboptimal target temperature based on the guide reference. [1] We performed a pilot study for choosing the optimal wavelength of LED for duodenal photobiomodulation (PBM). For this, experimental energy settings including 630nm, 850nm, dual (630nm/850nm), and RFA were evaluated in 1-week after duodenal intervention via gastrostomy using triangular shaped LED catheter with 2-illuminated dimension. (***Supplementary Figure 1A***) The RFA probe and LED catheter were inserted into the duodenum through the incised stomach near to the pyloric sphincter. The 5cm length of the first part of duodenum was irradiated as follows the energy setting protocol. (***Supplementary Table 1***) For the evaluation of thermal injury in the duodenal wall, an infrared camera (FLIR E6-XT; FLIR Systems Inc., OR, United States) was applied for thermal images in sham control, RFA, and each PBM group during procedure. (***Supplementary Figure 2***) Based on the optimal results of duodenal LED PBM in 1-week follow-up study, the experimental energy settings including 630nm and dual-wavelength (630nm/850nm) were evaluated in 4-week after duodenal LED PBM. In the 4 weeks follow-up study, we performed the LED PBM using the catheter with four illuminated dimensions so as to irradiate the whole surface of duodenal mucosal. (***Supplementary Figure 1B***)

Multiplex immunofluorescence staining:

Multiplex IHC stain, scan and analysis were performed on prismCDX Co.,Ltd (Gyeonggi-do, Korea). 3-μm sections of specimens were cut from formalin-fixed paraffin-embedded (FFPE) blocks. Slides were heated for at least one hour in a dry oven at 60℃, then followed by multiplex immunofluorescence staining with a Leica Bond Rx™ Automated Stainer (Leica Biosystems). The list of the antibody and fluorophore used is summarized in the table. Briefly, the slides were dewaxed with Leica Bond Dewax solution (#AR9222, Leica Biosystems), followed by antigen retrieval with Bond Epitope Retrieval 2 (#AR9640, Leica Biosystems) for 30 minutes. The staining proceeds in sequential rounds of blocking with antibody diluent / block (ARD1001EA, Akoya Biosciences), followed by primary antibody incubation for 30 minutes and Goat Anti-Rabbit IgG H&L (HRP polymer) (ab214880, Abcam) incubation for 10 minutes. Visualization of antigen was accomplished using tyramide signal amplification (Akyoa Biosciences) for 10 minutes, after which the slide was treated Bond Epitope Retrieval 1 (#AR9961, Leica Biosystems) for 20 minutes to remove bound antibodies before the next step in the sequence. The process from the blocking step to the antigen retrieval step is repeated for every antibody staining. Nuclei were stained with DAPI (62248, Thermo Scientific) for counterstaining after the last round of antigen retrieval. The slides were coverslipped using ProLong Gold antifade reagent (P36935, Invitrogen).

Multispectral imaging and analysis:

Multiplex stained slides were scanned using the Vectra Polaris Automated Quantitative Pathology Imaging System (Akoya Biosciences) at 20x magnification. Representative images for training were selected in Phenochart (Akoya Biosciences), and an algorithm was created in the inForm Image Analysis software (Akoya Biosciences). Multispectral images were unmixed using the spectral library in inFome software. Based on DAPI staining, each single cell was segmented and phenotyping was performed according to the expression compartment and intensity of each marker. After designating the region (ROI, region of interest) to be analyzed on the tissue slide, the same algorithm created in this way was applied and batch-running. The exported data is consolidated and analyzed in R software using the phenoptr (Akoya Biosciences) and phenoptrReport (Akoya Biosciences) packages.

To measure spatial cell to cell distance, nearest neighbor analysis was used to calculate the distance between two cells located closest to each other, and count within analysis was used to calculate the percentage of other cell types within a few 15, 30μm radius of one specific cell.

Reference

1. Cho JH., et al., Safety and effectiveness of endobiliary radiofrequency ablation according to the different power and target temperature in a swine model. J Gastroenterol Hepatol. Feb;32(2):521-526. 2017

Supplementary Table S1. Summary of parameters used for the experiments.

	Group	wavelength [nm]	Cross-section shape of catheter	LED chip x illuminating surface *	Time [s]	Power [W]	Energy [J]	Irradiance [mW/cm²]	Energy density [J/cm²]
1-week follow-up	LED (630)	630	triangular shape	10 x 2	600	0.018	10.8	3.7	2.22
	LED (850)	850	triangular shape	10 x 2	110	0.093	10.23	32.72	3.60
	LED (630/850)	630	triangular shape	10 x 1	500	0.018	9	3.7	1.85
		850		10 x 1		0.023	11.5	7.7	3.85
	RFA	-	round shape		3	7	-	-	-
4-week follow-up	LED (630)	630	square shape	10 x 4	600	0.036	21.6	3.7	2.22
	LED (630/850)	630	square shape	10 x 2	500	0.036	18	3.7	1.85
		850		10 x 2		0.046	23	7.7	3.85

* The one side of illuminating surface includes 10 LED chips

Supplementary Table S2. Raw data including mean and standard deviation (SD) from the 1-week follow up pilot study.

		Control (sham)		LED (630nm)		LED (850nm)		RFA		LED (630/850nm)	
		mean	SD	mean	SD	mean	SD	mean	SD	mean	SD
Figure 1C	Collagen deposition [%]	0.16	0.13					0.24	0.15	0.01	0.01
Figure 1D	Body weight gain [%]	6.58	6.90	8.50	5.32	10.06	8.28	3.40	3.21	0.63	1.92
	Food intake [g]	85.40	22.20	75.00	8.00	76.00	11.20	72.54	10.82	73.00	10.10
Figure 1E	AST (0W)	113.24	42.06							89.82	11.47
	AST(1W)	145.04	61.08							90.34	32.84
	ALT (0W)	50.44	16.49							45.80	6.41
	ALT (1W)	90.98	72.49							41.92	18.33
	Cholesterol (0W)	68.36	7.24							67.00	3.24
	Cholesterol (1W)	58.58	4.21							59.22	4.11
	ALP (0W)	757.18	105.03							843.02	82.51
	ALP (1W)	581.00	100.35							537.12	35.74

Supplementary Table S3. Raw data including the mean and standard deviation (SD) from the 4-week follow-up study.

		Control		Control (sham)		LED (630/850nm)	
		mean	SD	mean	SD	mean	SD
Figure 2C	Body weight gain [%]	18.68	5.67	21.98	4.24	20.02	3.33
	Food intake [g]	428.10	41.60	424.20	20.90	414.50	32.70
Figure 2D	AST (0W)	66.92	10.97	58.80	12.39	65.58	11.30
	AST(4W)	54.12	4.75	59.54	11.14	49.77	10.53
	ALT (0W)	32.68	4.56	31.26	5.10	35.43	7.72
	ALT (4W)	29.22	4.11	27.02	2.30	25.30	3.06
	Cholesterol (0W)	88.94	9.03	77.52	4.39	80.93	5.60
	Cholesterol (4W)	76.38	11.70	75.20	2.10	73.17	3.14
	ALP (0W)	149.26	27.04	105.18	29.79	95.35	28.65
	ALP (4W)	114.30	57.00	126.18	86.63	140.17	70.13
Figure 3B	area of the β cell	0.41	0.15	0.75	0.42	0.89	0.17
Figure 3C	HOMA-IR (0W)	0.17	0.04	0.14	0.05	0.18	0.04
	HOMA-IR (1W)	0.19	0.08	0.08	0.04	0.08	0.03
	HOMA-IR (4W)	0.12	0.03	0.13	0.05	0.12	0.04
Figure 4A	GIP_NC	12.91	8.34	8.02	2.53	8.43	3.23
	GIP_PC	11.16	5.10	8.84	2.54	11.20	2.90
Figure 4B	GLP-1_NC	10.62	5.38	7.66	1.63	6.83	2.46
	GLP-1_PC	8.96	4.49	5.37	0.95	4.88	1.05
Figure 6A	insulin (0W)	100.00	0.00	100.00	0.00	100.00	0.00
	insulin (1W)	113.67	22.80	99.68	1.88	99.68	1.88
	insulin (4W)	91.78	6.36	98.99	5.44	98.99	5.44
	GLP-1 (0W)	100.00	0.00	100.00	0.00	100.00	0.00
	GLP-1 (1W)	84.12	20.48	90.65	8.82	90.65	8.82
	GLP-1 (4W)	137.87	57.96	96.34	7.36	96.34	7.36
	GIP (0W)	100.00	0.00	100.00	0.00	100.00	0.00
	GIP (1W)	76.79	11.67	95.79	10.71	95.79	10.71
	GIP (4W)	114.43	33.93	105.57	7.00	105.57	7.00

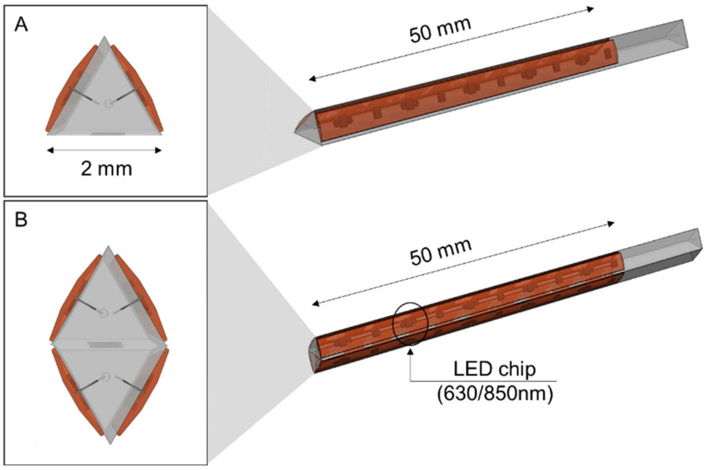

Supplementary Figure S1. Design of duodenal dual-wavelength photobiomodulation with light emitting diode (LED): (A) geometry of triangular catheter with 10 mini LED chips of two encapsulated surface using in 1 week follow-up study and (B) squared catheter with 10 mini LED chips of four encapsulated surface using in 4 week follow-up study.

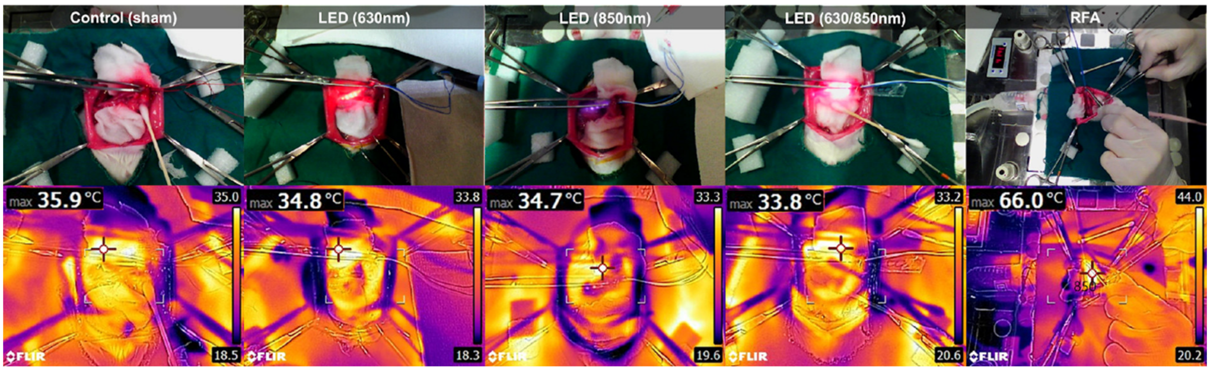

Supplementary Figure S2. Comparison of the therapeutic response in control and case groups including duodenal photobiomodulation with light emitting diode and RF probe by thermal images.

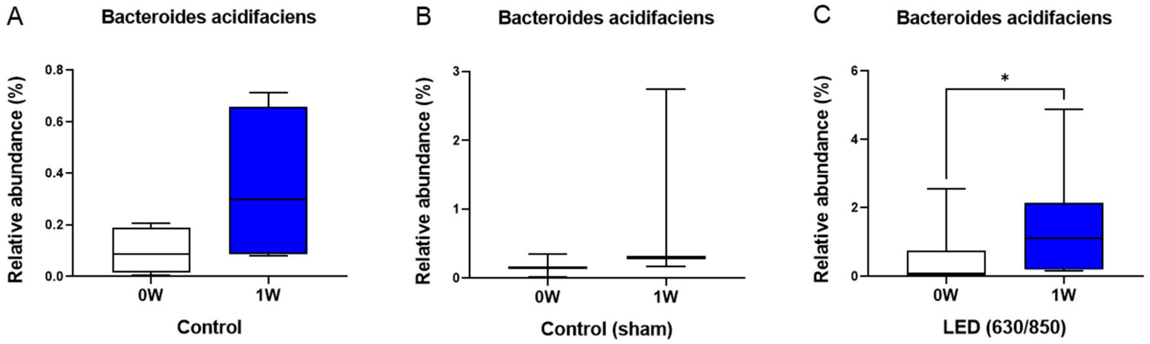

Supplementary Figure S3. Change of Bacteroides acidifaciens among the five enriched genera in 0- and 1- week in (A) a control group, (B) sham control group and (C) a group treated by duodenal dual-wavelength (630/850 nm) light emitting diode photobiomodulation.
